# Hyperperfusion profiles after recanalization differentially associate with outcomes in a rat ischemic stroke model

**DOI:** 10.1177/0271678X231208993

**Published:** 2023-10-24

**Authors:** Bart AA Franx, Geralda AF van Tilborg, Aladdin Taha, Joaquim Bobi, Annette van der Toorn, Caroline L Van Heijningen, Heleen MM van Beusekom, Ona Wu, Rick M Dijkhuizen, on behalf of the CONTRAST consortium

**Affiliations:** 1 Biomedical MR Imaging and Spectroscopy group, Center for Image Sciences, University Medical Center Utrecht and Utrecht University, Utrecht, The Netherlands; 2 Erasmus MC, Cardiovascular Institute, Thorax Center, Department of Cardiology, Rotterdam, the Netherlands; 3 Athinoula A Martinos Center for Biomedical Imaging, Department of Radiology, Massachusetts General Hospital, Charlestown, MA, USA

**Keywords:** Ischemic stroke, reperfusion, hyperperfusion, magnetic resonance imaging, sex factors, histology

## Abstract

Futile recanalization hampers prognoses of ischemic stroke after successful mechanical thrombectomy, hypothetically through post-recanalization perfusion deficits, onset-to-groin delays and sex effects. Clinically, acute multiparametric imaging studies remain challenging. We assessed possible relationships between these factors and disease outcome after experimental cerebral ischemia-reperfusion, using translational MRI, behavioral testing and multi-model inference analyses. Male and female rats (N = 60) were subjected to 45-/90-min filament-induced transient middle cerebral artery occlusion. Diffusion, T_2_- and perfusion-weighted MRI at occlusion, 0.5 h and four days after recanalization, enabled tracking of tissue fate, and relative regional cerebral blood flow (rrCBF) and -volume (rrCBV). Lesion areas were parcellated into core, salvageable tissue and delayed injury, verified by histology. Recanalization resulted in acute-to-subacute lesion volume reductions, most apparently in females (n = 19). Hyperacute normo-to-hyperperfusion in the post-ischemic lesion augmented towards day four, particularly in males (n = 23). Tissue suffering delayed injury contained higher ratios of hypoperfused voxels early after recanalization. Regressed against acute-to-subacute lesion volume change, increased rrCBF associated with lesion growth, but increased rrCBV with lesion reduction. Similar relationships were detected for behavioral outcome. Post-ischemic hyperperfusion may develop differentially in males and females, and can be beneficial or detrimental to disease outcome, depending on which perfusion parameter is used as explanatory variable.

## Introduction

With the advent of reperfusion therapy, most recently mechanical thrombectomy, stroke interventionalists received newfound opportunities to counteract proximal cerebrovascular occlusions.^1^ Yet despite these advances, more than half of acute ischemic stroke (AIS) patients lack meaningful improvement after intra-arterial treatment.^2^

Futile recanalization has been addressed in clinical literature; time-to-reperfusion,^3^ severity at admission, and sex^4^ are risk factors explaining variability in treatment success. However, multitudes of hypotheses implicate reperfusion aberrations, such as post-ischemic hyperperfusion,^5^ changes in capillary transit time variability,^6^ and incomplete microvascular reperfusion (IMR),^7^ as culprits that interact with the abovementioned factors, shaping the early course of the disease and chance of recovery. There is no consensus on causation behind lagging patient recovery rates and the role of post-ischemic hemodynamics in this process. Exhaustive characterization of post-ischemic hemodynamics would shed more light on how reperfusion associates with stroke outcome, such that avenues for therapeutic strategies or management may be discovered.

Brain imaging methods like magnetic resonance imaging (MRI), are instrumental in diagnosis of tissue injury and perfusion disturbances. Acute ischemic tissue, manifesting as cytotoxic edema and resulting in a reduction in the apparent diffusion coefficient (ADC) of tissue water, can be detected with diffusion-weighted MRI.^8,9^ Later, in the subacute phase, tissue necrosis, blood-brain barrier injury and vasogenic edema develop in the infarct area, causing water accumulation that results in local T_2_ prolongation, which can be detected with T_2_-weighted MRI.^8,9^ Reperfusion may lead to salvation of acutely ischemic tissue with reduced ADC and prevention of development of infarction with concomitant T_2_ prolongation.^10–12^ This means that salvageable tissue can be identified from the combination of acute diffusion-weighted MRI and subacute T_2_-weighted MRI.^11^ Similarly, tissue without acute ADC reduction but with subacute T_2_ prolongation after reperfusion would reflect delayed injury, while tissue with both acute ADC reduction and subacute T_2_ prolongation would represent the lesion core.

Longitudinal multiparametric imaging studies of AIS patients are scarce. Clinical data on post-stroke reperfusion beyond the acute phase (>24 hours post-AIS) have shown normal (normoperfusion), elevated (hyperperfusion) as well as markedly reduced (hypoperfusion) perfusion, which may exist on a continuum. As extensively reviewed recently,^13^ several lines of evidence suggest hyperperfusion may be associated with beneficial outcomes in patients, treated^14,15^ or untreated,^16,17^ while conversely, hyperperfusion has also been associated with hemorrhagic transformation in patients who received reperfusion therapy.^18^ Perhaps unsurprisingly, normoperfusion associates with beneficial outcome in patients treated by mechanical thrombectomy.^15^ Lastly, acute post-recanalization hypoperfusion accompanies unfavorable outcomes but appears to be less prevalent than hyperperfusion in patient studies.^13^

Although hemodynamic aberrations may already develop within hours after reperfusion therapy, few perfusion studies have been executed in patients as post-recanalization imaging is not standard of care. Translational studies in animal stroke models can overcome procedural challenges present in the clinic, with the added benefit of providing tightly controlled conditions and imaging time points. Indeed, preclinical MRI studies in animal stroke models have characterized both early and late perfusion deficits,^19,20^ but the question how early post-ischemic hemodynamics foreshadow stroke outcome beyond the acute phase remains largely unanswered. Stroke outcomes may depend on variable occlusion-to-recanalization times in conjunction with sex,^4^ the latter of which is often neglected,^21^ even though differences in post-ischemic perfusion may underlie these effects and could partly explain variation in futile recanalization. Lastly, while CBF has historically enjoyed the most attention in the literature, cerebral hemodynamics are comprised of several other key features such as cerebral blood volume (CBV) and mean transit time (MTT), which may uniquely contribute to explain stroke outcome.^22^

Here, transient middle cerebral artery occlusion (tMCAO) by intraluminal filament was employed as a model of ischemic stroke followed by mechanical recanalization^23^ in male and female rats, followed by a longitudinal multiparametric MRI paradigm. Using dynamic susceptibility contrast-enhanced perfusion-weighted imaging (DSC-PWI), we extensively characterized the magnitude and spatial heterogeneity of cerebral perfusion aberrations before and after recanalization in diffusion- and T_2_-weighted MRI-derived regions-of-interest (ROIs). The definition of these ROIs was guided by tissue fate and validated histologically. Additionally, we aimed to identify hyperacute post-recanalization cerebral perfusion parameters that associate with acute-to-subacute lesion volume change and functional outcome.

## Methods

Animal procedures and experiments were approved by the Animal Experiments Committee of the University Medical Center Utrecht and Utrecht University, and were performed in accordance with the guidelines of the European Communities Council Directive. Animal experiments were performed and reported according to the ARRIVE guidelines. Raw imaging data has been made available for use on Zenodo (DOI: 10.5281/zenodo.8139567).

### Animals

Male and female adult Sprague-Dawley rats (N = 60, 11–13 weeks) from Charles-River were used, of which forty-two (nineteen females) were included in the final analysis. Rats were housed in single-sex pairs under standard conditions with light/dark cycle of 12/12 hours (light on from 07:00 till 19:00), and *ad libitum* access to food and water. Rats were randomly assigned to right tMCAO of either 45 or 90 minutes per cage group by random number generation. Researchers could not be blinded to sex or occlusion time. Commonly used occlusion times of 45 and 90 minutes were chosen as longer ischemic conditions produce large lesion volumes close to the expected upper bound,^24^ which is arguably less representative of patient cases eligible for treatment under current guidelines.^25^ Animals were excluded a-priori if an ischemic lesion was not detected in the right striatum or neocortex, or ischemic lesion size was insufficient (<30 µL). This led to the exclusion of eleven animals. An additional seven rats were excluded due to surgery complications. There was no premature mortality.

### Animal anesthesia and monitoring

Before surgery and MRI experiments, rats were anesthetized with 4% isoflurane for endotracheal intubation, followed by mechanical ventilation with 2% isoflurane in medical air:O_2_ (4:1). Before each MRI session, animals received ophthalmic cream (Duratears™ Z, Alcon, Switzerland) on their eyes in addition to 10 mg/kg subcutaneous lidocaine (Xylocaine 5%, AstraZeneca, Sweden) in the throat area prior to surgery. End-tidal CO_2_ was continuously monitored with a capnograph, guiding ventilation tidal volume and breathing rate. Body temperature was maintained at 37 ± 0.5 °C with a feedback-controlled heating pad. During MRI experiments, blood oxygen saturation and heart rate were continuously monitored. Rats were subcutaneously injected with 0.9% NaCl (1 ml/100g) postoperatively and after MRI sessions to replenish lost fluids. Rats were placed on heating mats for the first day(s) after surgery. Body weight was measured daily before and after surgery. Predefined conditions for humane endpoints were not exceeded. Additional 0.9% NaCl injections were administered if deemed necessary by visual inspection of hydration status (1 ml/100g, subcutaneous).

### Experimental work flow and ischemic stroke model

Three days before surgery, baseline behavioral tests were conducted as described below. At the day of surgery, animals were anaesthetized as described above, and tMCAO was induced as described by Zea Longa et al.^26^ Briefly, the right common carotid artery (CCA), internal carotid artery (ICA) and external carotid artery (ECA) were exposed and dissected. The CCA was temporarily ligated while the ECA was cauterized and arteriotomized. The ECA was reflected along the ICA such that a silicon-tipped nylon filament (4-0, Doccol Corporation, USA) could be advanced until resistance was felt. The filament was left occluding the MCA for 45 or 90 minutes. Meanwhile, the wound was temporarily closed with Tergaderm (3 M, USA) and, without discontinuation of anesthesia, the animal was transferred to the MRI scanner for the first examination. Afterwards, the filament was slowly removed and a 2 mg/kg intra-incisional injection of bupivacaine (Levobupivacaine 0.25%, Fresenius Kabi, Germany) was administered before the wound was closed. The animal was transported back to the MRI system for a second examination, after which it was allowed to recover. After four days, behavioral assessment was repeated and animals were re-anesthetized for the final MRI examination, after which they were sacrificed by funnel-freezing^27^ for further histologic examination (see below).

### Behavioral testing

A sensorimotor deficit score (SDS) was calculated from an adapted battery of sensorimotor tests^28^ several days before stroke induction and four days later before the final MRI session. Briefly, animals were scored consecutively on spontaneous exploratory mobility and gait disturbance, lateral resistance (when pushed sideways), whisker-guided forelimb placing, forelimb grasping- and strength on a horizontal bar, and postural signs when being held by the base of the tail. Total SDS ranged from 0 (no deficit) to 22 (maximum deficit). Test items are listed in Supplementary Table I. Researchers could not be blinded to sex or occlusion time.

### MRI

MRI experiments were conducted on a horizontal bore 9.4 T MR system (Varian, Palo Alto, CA, U.S.A.), equipped with a 20.5-cm gradient able to generate 400 mT/m. A Helmholtz volume coil (Ø 80 mm) and inductively coupled surface coil (Ø 25 mm) were used for signal transmission and detection, respectively. Anesthetized rats were placed in a MR-compatible stereotactic holder and restrained with a headset and tooth bar and mechanically ventilated (see *Animal anesthesia and monitoring*). The MRI protocol included diffusion-weighted (8-shot multi-slice spin-echo echo planar imaging (SE-EPI); repetition time (TR)/echo time (TE) = 2000/31 ms; b = 0, 1454 s/mm^2^), and T_2_-weighted imaging (8-shot multi-slice SE-EPI; TR/[TE] = 3000/[30, 50, 80, 190] ms), both with a field-of-view (FOV) set to 33.75 × 33.75 × 15.00 mm^3^, containing twenty-five contiguous 0.6 mm thick coronal slices with 224 × 224 matrix size, resulting in 150 × 150 × 600 μm 3 voxel size. In addition, dynamic susceptibility contrast-enhanced perfusion-weighted MRI (DSC-PWI) was executed with a gradient echo (GE) EPI sequence (2 D multi-slice GE-EPI; TR/TE = 164/13 ms, FOV 31.2 × 31.2 × 10.8 mm^3^; six contiguous 1.2 mm thick coronal slices containing a 64 × 64 data matrix with a 0.6 mm slice gap), combined with an intravenous bolus injection of Gd-DTPA (Gadobutrol, Bayer Healthcare, Germany) (0.35 mmol/kg), injected at the 180^th^ image. Precursory 3 D GE and SE images were acquired for registration purposes (2 D multi-slice GE-EPI; TR/TE = 164/13 ms, FOV 31.2 × 31.2 × 14.4 mm^3^; eight contiguous 1.2 mm thick coronal slices containing a 64 × 64 data matrix with a 0.6 mm slice gap). A few individual DSC-PWI experiments were excluded due to Gd-DTPA injection failure.

### Image processing

All MR images were processed using FSL 5.0. Images were corrected for inhomogeneities^29^ and brain extraction was performed with FSL’s Brain Extraction Tool.^30^ Image registration was performed with FSL FLIRT^31^ and additionally with the FSL Non-linear Image Registration Tool (FNIRT) if necessary. For each subject, the unweighted (b = 0) image from the diffusion-weighted MRI scan acquired during MCAO served as the internal anatomical reference (hereafter: native space), to which each subsequent b0 image from different time points was registered, to aid the alignment of masks to and from ADC-, T_2_- and perfusion maps (see *Semi-automatic lesion thresholding* and *Region-of-interest (ROI) analysis*).

Maps of the mean apparent diffusion coefficient (ADC), calculated by monoexponential fitting of the diffusion-weighted imaging data, were obtained by averaging three ADC maps acquired with diffusion-sensitive gradients applied along the cardinal directions x, y or z. Quantitative T_2_ maps were calculated by derivative-based regular least squares fitting of complex-valued data.^32^ Maps of cerebral blood flow (CBF), cerebral blood volume (CBV) and mean transit time (MTT) were calculated by circular deconvolution of tissue concentration curves using an arterial reference curve obtained from the contralateral hemisphere.^33^ Oscillation index regularization was set to 0.17. CBV was calculated by numeric integration of the tissue concentration curve, truncated at the 400^th^ image to minimize contrast agent recirculation effects.

### Semi-automatic lesion thresholding

Diffusion-weighted images acquired during occlusion (acutely) and T_2_-weighted images acquired after four days (subacutely), were aligned (through native space) to a template rat brain, which contained a high-resolution parcellated image of the Paxinos and Watson rat brain atlas,^34^ which could then be back-transformed to the original data. Using the brain atlas aligned with original parametric maps in subject space, ADC and T_2_ values were extracted from the contralateral MCA territory: i.e., the primary somatosensory cortex (S1FL), secondary somatosensory cortex (S2) and caudate putamen (CPu). Voxels were then thresholded to exclude CSF, after which their mean (*x̄*) and standard deviation (*sd*) was calculated, to be used as reference values for the lesion masking procedure. Acute ischemic tissue was defined by an ADC at least 2 standard deviations lower than the mean contralateral ADC ( $<\bar{x}-2sd$ ), while subacutely, voxels were considered lesioned if the T_2_ exceeded the contralateral mean by more than 2 standard deviations ( $>\bar{x}+2sd$ ). Lesion masks were manually noise-corrected.

### Region-of-interest (ROI) analysis

To mitigate confounding effects of subacute brain edema, which in turn may cause midline shift, T_2_-based lesion masks from the subacute phase were non-linearly aligned to images in each subject’s native space. In native space, the following ROIs and their contralateral homologues were defined, in addition to the acute ischemic tissue and subacute infarct areas: 1) ischemic tissue that appeared normal at day four and had thus been able to recover, i.e. salvageable tissue = acute ischemic area – subacute infarct area, 2) irreversible acute ischemic injury that proceeded to infarction, i.e. lesion core = acute ischemic area – salvageable tissue, 3) post-recanalization injury, i.e. delayed injury = subacute infarct area – acute ischemic area. Contralateral homologues were obtained by aligning mirrored copies of ROIs to the source image in native space.

ROI volumes were expressed as percentage of the hemispheric volume (calculated in native space using ROI mask volumes). The percent change in acute-to-subacute lesion volume change was calculated from the difference between the T_2_-derived hemispheric volume of the subacute infarct and the ADC-derived hemispheric volume of acute ischemic tissue: 100 × (T2_lesionVolume_ – ADC_lesionVolume_)/ADC_lesionVolume_.

Lesion masks and contralateral homologues were then aligned to each ADC-, T_2_- and perfusion map (CBF, CBV and MTT). For perfusion indices, average perfusion values in the (post-)ischemic area were calculated and expressed as a percentage of the average value in its contralateral homologue, producing a single relative regional perfusion index (rrCBF, rrCBV and rrMTT), eliminating inadvertent within- or between-subject variability from slight bolus injection speed deviations.

**Figure 1.**
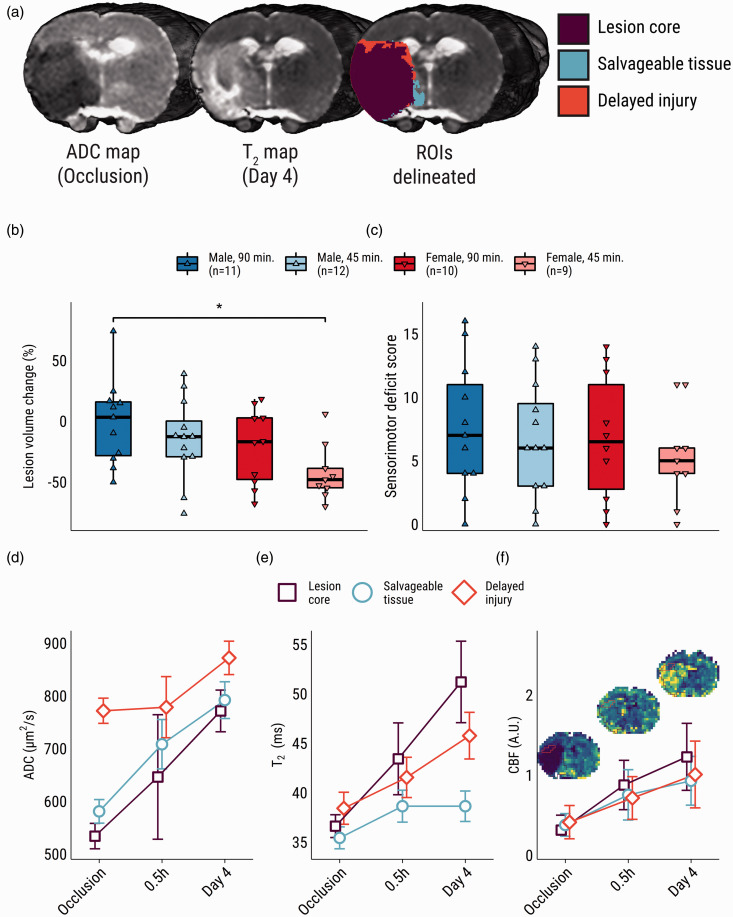
Outcomes of cerebral ischemia-reperfusion on MRI- and behavioral measures. (a) Representative examples of an ADC-map during MCAO, a T_2_-map 0.5 h after recanalization, and subsequent ROI-delineation. (b) Box- and violin plots of acute-to-subacute lesion volume change. (c) Subacute sensorimotor deficit scores. (d) ROI-specific time course of mean (±SD) ADC, (e) and T_2_. (f) ROI-specific time courses of CBF with example CBF-maps and ROI overlays of the representative subject shown in panel a. ADC: apparent diffusion coefficient; ROI: region-of-interest; CBF: cerebral blood flow. *p < 0.05.

In addition to relative perfusion magnitudes, we characterized spatial heterogeneity of perfusion per ROI (see Figure 1(f) and Supplementary Figures I and II for examples). Delayed injury or salvageable areas were often small and close to feeding arteries or CSF (Figure 1(f); example CBF-maps), frequently resulting in non-physiological perfusion values contaminating averages from these small ROIs. To reduce the effects of these errors, we applied a data binning strategy. First, each voxel of a particular perfusion map was repercentaged to an internal control value, here the contralateral median. Next, we thresholded these repercentaged perfusion values from continuous to nominal scale. Using ±15% thresholds, based on a recent observation that perfusion asymmetry of >15% can be clinically significant,^35^ voxels were binned as “hyperperfused” when they exceeded 115% of the contralateral median, or “hypoperfused” when they dropped below 85%. With MTT, the classification was reversed: short (<85%) and long (>115%) MTT values signified hyper- and hypoperfusion, respectively. Voxels within the 85-115% range were regarded “normoperfused”. Voxel classes per ipsilesional and contralesional ROI (i.e. core, salvageable tissue or delayed injury) were summed and expressed as a fraction of the total voxel count in the ROI that voxel belonged to.

### Histology

Funnel-frozen brains were processed for histological analysis.^27^ We selected nine male brains from the 45- and 90-minute occlusion groups that showed well-defined ROI parcellation that was large enough for alignment and histological scoring to validate our MRI-driven lesion parcellation method. Male-female comparisons were not part of this analysis. Frozen brains were sectioned in coronal slices of 3-mm thickness. The slice of interest was fixed in 4% buffered paraformaldehyde and a sucrose gradient and cut with a cryomicrotome to obtain 10-µm thick histological sections, which were then stained with hematoxylin-eosin (HE). HE-stained sections were digitized at 80x magnification (NanoZoomer. Hamamatsu, Japan) and manually aligned to the *in vivo* MRI images, based on visual assessment and the Paxinos rat brain atlas.^34^ Only HE-stained sections that achieved a good alignment with the plane of the MR images were analyzed, which was a decision based on expert consensus between pathologists and imaging scientists on the team.

The histological evaluation of the brain specimens consisted of histopathological scoring of 0.15 mm^2^ areas within the different ROIs defined in the MRI analysis: lesion core, salvageable tissue and delayed injury and contralateral control areas. For all ROIs, both cortex and caudate putamen regions were studied. The scoring included the evaluation of the following histological features of brain infarction: nucleus abnormalities (karyolisis, karyopyknosis or karyorrhexis), loss of nuclei, presence of red neurons (cytoplasmatic eosinophilia), vacuolization (neuropil spongiosis), loss of eosinophilia, extracellular edema, and hemorrhage. All categories were semi-quantitatively scored from 0 to 3, with 0 indicating absence of the histological feature, and 3 indicating a high presence of the histological feature. Scoring was performed by two independent researchers (JB, HB) blinded to the ROI designation. When scores for an area differed between both researchers, the area was analyzed again (still blinded), and a consensus score was given. Histopathalogical events were counted in at least six ROIs from different subjects. Scores were accumulated per ROI and compared.

### Statistical analyses

We conducted an *a priori* power analysis with GPower (v3.1.9.7) to detect a significant increase in R^2^ if at least one predictor was added to the base model, here a cerebral perfusion variable. Because no previous linear models that assessed the relationship between occlusion duration and sex with dependent variable lesion volume change are available, we chose Cohen’s *f* ^2^ = 0.35, which can be justified given that effect sizes found in preclinical research are typically large. Consequently, at an α-level of 0.05 and 1 – β = 0.8, a sample size of 40 was required.

Assumptions for each parametric test, such as the supposed distribution of the response variable (i.e., normal, beta or Poisson), homogeneity of variance and normality of residuals, were checked but no violations were encountered. Bonferroni-correction was applied for post-hoc testing unless otherwise specified. Values are shown as mean ± SD unless otherwise specified.

For histology, the distribution of the median scores across lesion core, salvageable tissue and delayed injury was analyzed with a Kruskal-Wallis test. Dunn’s multiple comparisons test was performed for post-hoc comparison of salvageable tissue and delayed injury areas versus the lesion core.

To analyze differences in perfusion magnitude over time, linear mixed-model analysis was performed for each relative regional perfusion index. rrCBF, rrCBV and rrMTT were compared per time point (occlusion vs. 0.5 h vs. day four), per occlusion time (tMCAO: 45 vs. 90 min) and sex (M vs. F). Subject (i.e. rat) was set as random effect. Residual degrees-of-freedom were restricted by Kenward-Roger approximations.

To analyze spatio-temporal heterogeneity of cerebral perfusion within each ROI, binned perfusion voxels (hypoperfused or hyperperfused) were expressed as a fraction of the total ROI volume for both ipsilateral delayed injury areas and the contralateral homologue. These fractions were analyzed over time using a generalized linear mixed model from the beta family with a log-link function. Hemisphere (ipsilateral vs. contralateral), ROI (lesion core vs. salvageable tissue vs. delayed injury) and time (occlusion vs. 0.5 h vs. day four) were fixed-effect terms. Subject (i.e., rat) was set as a random effect.

To assess a relationship between lesion evolution and immediate post-ischemic reperfusion, the following predictors were regressed against lesion volume change: tMCAO (45 vs. 90 minutes), sex (M vs. F), and their interaction as fixed cofactors; relative regional perfusion indices (rrCBF, rrCBV, rrMTT) as co-variables. Lesion location (diencephalic, subcortical or cortical) and hemispheric volume of the acute ischemic lesion volume were included to control for variability in lesion etiology. Continuous variables were scaled and centered. Next, a multi-model inference approach was applied to evaluate the predictive value of perfusion indices by subsetting the full model. The MTT is, according to the central volume theorem, the quotient of CBV and CBF, hence not independent of rrCBV and rrCBF, therefore including all three terms will not yield the most parsimonious or interpretable model. To determine whether there was a set of perfusion variables that best explained lesion change after recanalization, models were refit with four parameter configurations where rrMTT was not included if rrCBF or rrCBV was already present. Other explanatory and nuisance parameters remained fixed. The same strategy was applied to analyze functional outcome quantified by SDS but with a generalized linear model regression of the Poisson family with log-link function. Models were ranked by second-order corrected Akaike Information Criterion (AICc) for small sample sizes.

Statistical analyses were performed in R 4.0.2 using packages *tidyverse,*^36^ *lme4,*^37^ *glmmTMB,*^38^ *emmeans,*^39^ *car,*^40^ *MuMIn*^41^ and *stargazer.*^42^ Histological data was analyzed using Prism (version 8.0.0, GraphPad Software, La Jolla, California, USA).

## Results

### MRI-based detection of spatiotemporal lesion changes reflect various tissue fates after intraluminal tMCAO

tMCAO induced ADC decrease (Figure 1(a)) within the lesion core and salvageable tissue during occlusion, ultimately resulting in T_2_ prolongation in the core and delayed injury areas, but not in salvageable tissue (Figure 1(d), Figure 1(e)). Average ROI-specific time courses for CBF were similar but showed some intra-hemispheric variability as seen in example CBF-maps (Figure 1(f), and Supplementary Figures I and II). The hemispheric volume of acute ischemic tissue was similar between groups (0.15 ± 0.10 (90 min, males), 0.18 ± 0.11 (45 min, males), 0.14 ± 0.08 (90 min, females), 0.20 ± 0.10 (45 min, females)). See Supplementary Table II for descriptive statistics of all ROI volumes. Acute-to-subacute lesion volume reduction was larger in females compared to males (−31.4 ± 29.1% vs. −8.2 ± 34.4%, *p* = .02). Post-hoc comparisons showed significantly higher acute-to-subacute lesion shrinkage in the female 45-minute occlusion group as compared to the male 90-minute occlusion group (*p* = .03; Figure 1(b)). No group-effects were detected in a two-way ANOVA of the SDS at day four (Figure 1(c)).

**Figure 2.**
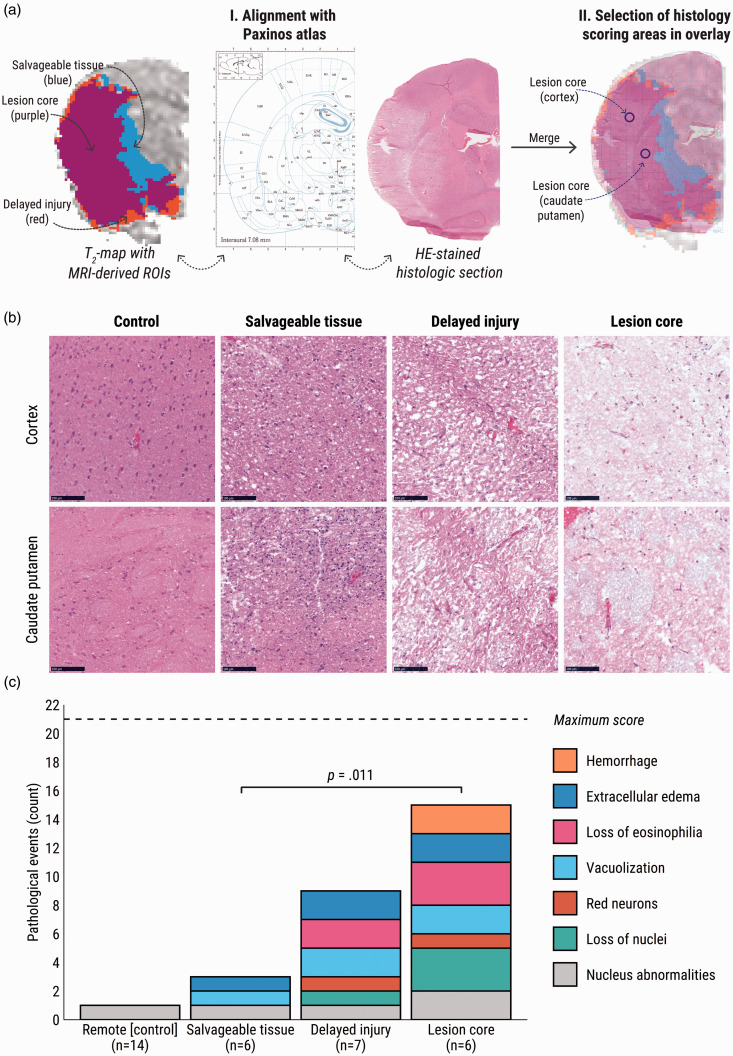
Histopathological scoring of MRI-derived ROIs at four days after tMCAO. (a) I: depiction of the process of alignment of MR images, including MRI-derived ROIs, with histology sections for histopathological scoring. Correct alignment of the MR image and the histological section was confirmed by comparison of anatomical structures using the Paxinos atlas of the rat brain. II: merging of the MR and histological images facilitated specific scoring of regions in the cortex and caudate putamen. Circular areas of 0.15 mm^2^ on the HE-stained sections were selected in the center of the ROIs (the “lesion core” in this example), for both cortex and caudate Continued.putamen, as guided by the anatomical atlas. (b) Representative microscopy images of hematoxylin-eosin-stained tissue in ROIs within the cortex (upper row) and the caudate putamen (lower row). Scale bar represents 100 µm. (c) Median of pathological events counted per ROI and remote area; “n” indicates the number of MRI-derived ROIs that were sampled for events. The salvaged tissue was more akin to the control area, scoring significantly lower than lesion core areas. All histological images were blued, and exposure was adjusted to improve readability. Representative images of the studied histopathological features at higher magnification are shown in Supplementary Figure III.

Histological analysis (Figure 2(a)) revealed different patterns of tissue damage among the MRI-derived ROIs of lesion core, salvageable tissue, and delayed injury (Figure 2(b)). Core areas presented the highest histopathological scoring with a moderate-to-high presence of all relevant pathological features of infarction. Delayed injury also featured characteristics of infarcted tissue but with slightly lower scores for nucleus abnormalities, loss of nuclei, eosinophilia and hemorrhage (Figure 2(c), N.S.). In contrast to delayed injury areas, significantly less pathological features were counted after reperfusion in the salvageable tissue as opposed to the lesion core (Figure 2(c), *p* = .01). Besides minimal presence of nucleus abnormalities, which could also be observed in the remotely-located control areas, the salvageable tissue only displayed some vacuolization and extracellular edema.

### Hyperperfusion emerges after recanalization

**Figure 3.**
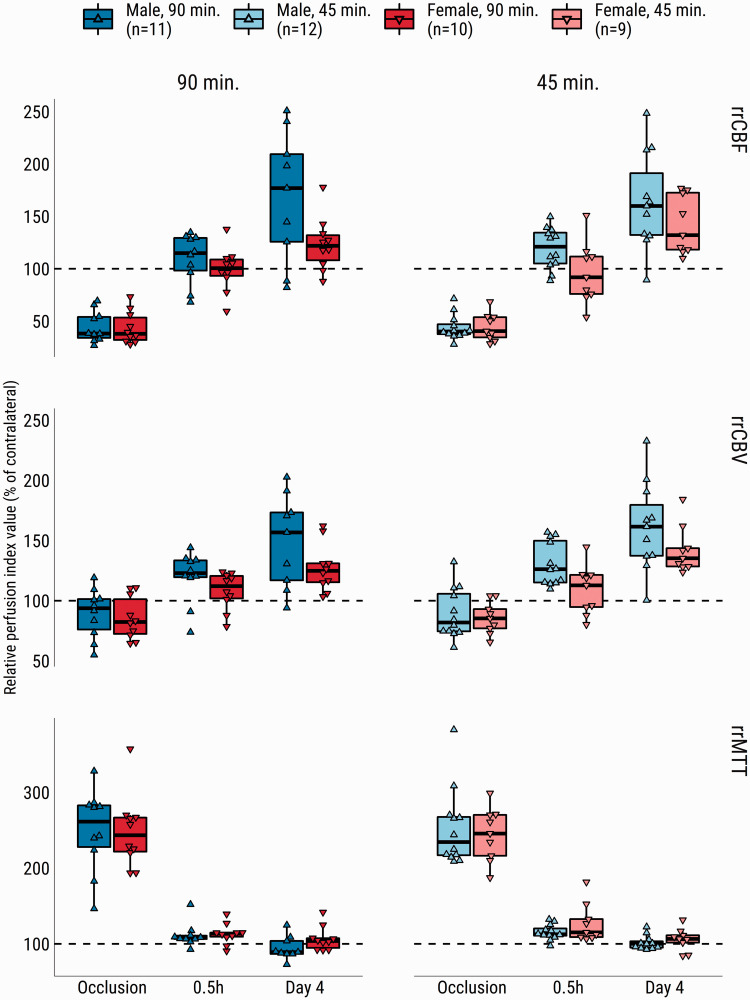
Relative regional cerebral blood flow (rrCBF), -cerebral blood volume (rrCBV) and -mean transit time (rrMTT) in the (post-)ischemic zone of male and female rats during 90- or 45- min MCAO and at 0.5 h and four days after recanalization. Values are expressed as a percentage of the contralesional mean from a homologous area. Dashed line indicates hypothetical normal level per hemodynamic parameter. Representative images of the perfusion indices are shown in Figure 1(f), and Supplementary Figures I and II.

Profound changes in relative regional cerebral perfusion were evident in the (post-)ischemic tissue throughout the experiment (Figure 3). There were large reductions in relative regional cerebral perfusion (i.e., normalized by contralateral hemisphere) during MCAO. After recanalization until the end of the experiment, the rrCBF and rrCBV values (Figure 3, first and second row, respectively) progressively shifted upward. Prolonged rrMTT values during occlusion, on average, returned to baseline at 0.5 h, and were shortened further at day four (Figure 3, third row). There were strong main effects for *time* for all relative regional perfusion indices (Supplementary Table III). Significant main effects for *sex* were found for rrCBF and rrCBV, while effects of *occlusion duration* did not reach significance. An interaction effect was detected for rrCBF, suggesting that flow differences between imaging time points were unequal between sexes (*p* = .035), however this interaction effect did not survive post-hoc testing. Post-hoc comparisons for main effects are shown in Supplementary Table IV.

After perfusion maps were repercentaged to their respective internal control value, empirical cumulative density functions of all voxels within the (post-)ischemic area were calculated for each perfusion parameter to exemplify the global perfusion shift for males (Supplementary Figure I) and females (Supplementary Figure II). These distributions also show that post-stroke perfusion is not homogeneous: mixtures of increased and decreased perfusion can be detected in (post-)ischemic areas.

### Delayed injury areas display irregular reperfusion patterns

**Figure 4.**
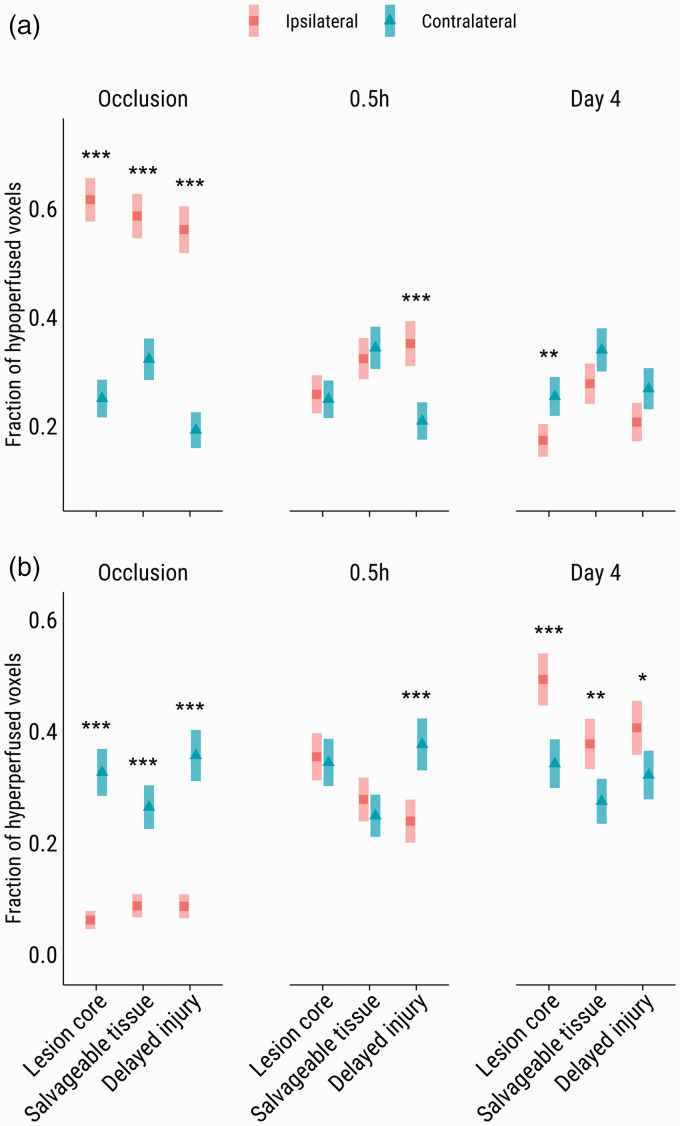
Estimated marginal means (±95% CI) of the fractions of abnormally perfused voxels per ipsi- and contralesional ROI on CBF maps. Voxels were classified as (a) hypoperfused or (b) hyperperfused, averaged over males, females and occlusion duration. ^#^*p* < 0.10, *p < 0.05, ***p < 0.001. Representative CBF maps are shown in Figure 1(f), and Supplementary Figures I and II.

Following binning of repercentaged CBF values to ±15% thresholds, the majority of voxels in each ROI was classified as normoperfused after recanalization. Modeling the fractions of hypo- and hyperperfused voxels per ROI revealed that significantly larger fractions of hypoperfused voxels were found in delayed injured areas (Figure 4(a), *p* < .001) compared to their contralateral control areas at 0.5 hours post-recanalization. Similarly, the fraction of hyperperfused voxels was comparatively lower in the delayed injured areas (Figure 4(b), *p* < .001). By day four, any hypoperfusion had largely disappeared from these ROIs. Conversely, there were significantly higher fractions of hyperperfused voxels in the lesion core (*p* < .001), salvaged tissue (*p* < .001) and delayed injury area (*p* < .05).

### Early reperfusion profile associates with lesion volume change and functional outcome

Linear regression analysis was performed to estimate the effects of post-recanalization cerebral perfusion on stroke outcome, defined as lesion volume change or functional performance (SDS). First, in the full model, relative regional perfusion parameters (rrCBF, rrCBV and rrMTT) were regressed against *lesion volume change*, alongside occlusion duration and sex, while controlling for ADC-derived acute ischemic lesion volume and lesion location (F(9,41) = 6.56, *p* < .001, adj. R^2^ = .56). All terms but the acute ischemic volume, rrMTT and interaction term had independent effects on lesion volume change. The full model was then subsetted using the four principal configurations of perfusion indices. The best performing model contained independent predictors rrCBF (β = 15.5, *p* = .02) and rrCBV (β = −16.8, *p* = .01) with an AICc difference of −2.3 compared to the model without perfusion indices (Table 1). Interestingly, signs of the model predictors indicated that higher post-recanalization rrCBF contributes to lesion growth, while lesion shrinkage is preceded by high rrCBV.

**Table 1.** Coefficients and performance measures for models with different configurations of perfusion parameters regressed against lesion volume change and ranked by performance (AICc).

	Models (ranking)					
	rrCBF & rrCBV (1)	rrMTT (2)	*¬DSC-PWI* (3)	rrCBV (4)	rrCBF (5)	*NULL* (6)
*Model coefficients (standardized β (±95 CI))*						
rrCBF	20.4*			3.6		
	**(5.6, 35.2)**			(−5.3, 12.6)		
rrCBV	−20.8*				−3.3	
	**(**−**36.1,** −**5.5)**				(−12.5, 6.0)	
rrMTT		−7.1^#^				
		(−15.1, 1.0)				
tMCAO (90 min.)	26.2*	23.9^#^	30.9*	29.9*	31.1*	
	**(4.5, 47.9)**	(0.02, 47.8)	**(7.8, 54.1)**	**(6.5, 53.3)**	**(7.8, 54.5)**	
Sex (M)	28.7*	21.6^#^	27.2*	23.8*	30.5*	
	**(6.7, 50.7)**	(−0.6, 43.9)	**(5.3, 49.1)**	**(0.2, 47.4)**	**(6.5, 54.4)**	
Acute ischemic volume	9.4	10.4	12.3^#^	11.6^#^	12.5^#^	
	(−2.8, 21.6)	(−2.3, 23.2)	(−0.6, 25.3)	(−1.5, 24.7)	(−0.6, 25.5)	
Subcortical lesion	−19.7	−15.1	−13.8	−13.1	−15.4	
	(−45.3, 5.8)	(−41.5, 11.3)	(−40.9, 13.2)	(−40.4, 14.2)	(−43.1, 12.2)	
Diencephalic lesion	−31.2*	−29.0*	−28.0^#^	−26.1^#^	−30.2*	
	**(**−**57.8,** −**4.6)**	**(**−**56.3,** −**1.6)**	(−56.1, 0.1)	(−54.8, 2.5)	**(**−**59.1,** −**1.2)**	
tMCAO:Sex	−5.8	−2.8	−7.8	−5.8	−9.1	
	(−34.8, 23.2)	(−33.6, 27.9)	(−38.8, 23.3)	(−37.4, 25.7)	(−40.7, 22.4)	
*Model performance*						
AICc	389	390.9	391.1	393.7	393.8	408.5
F	3.92	2.95		0.63	0.48	
*p* (>F _¬_*_DSC-PWI_*)	0.03	0.1		0.43	0.49	
RMSE	19.9	21.3	22.2	22	22.1	33.5
Residual df	32	33	34	33	33	40
Adjusted R^2^	0.59	0.55	0.52	0.52	0.52	

*Note: Models were tested against the base model without perfusion indices (¬DSC-PWI),* *which included only tMCAO, sex and nuisance variables*

tMCAO: transient middle cerebral artery occlusion; rrCBF: relative regional cerebral blood flow; rrCBV: relative regional cerebral blood volume; rrMTT: relative regional mean transit time; AICc: corrected Akaike information criterion, RMSE: root mean square error. #p < .10; *p < .05.

Secondly, the same full set of predictors were regressed against the SDS (*Χ^2^*(8,39) = 72.4, *p* < .001, Nagelkerke R^2^ = .86). Again, models were refitted using the four configurations of perfusion indices and ranked. The best performing model differed −0.9 in AICc compared to the base model without perfusion indices and contained rrMTT (β = −0.17, *p* = .04), indicating that prolonged rrMTT after recanalization associated with improved functional outcome (Supplementary Table V).

Finally, we sought to provide some mechanistic explanation (albeit *ad hoc*) for the independent effects of cerebral perfusion on experimental stroke outcome described above. Therefore, in an exploratory analysis, we searched for relationships between any hyperacute cerebral perfusion parameters and cytotoxic (ADC) and vasogenic edema (T_2_) in the post-ischemic area. We detected but a single correlation between rrMTT and ADC at 0.5 h post-recanalization (r = 0.41, *p* = .008). We also found that this early post-ischemic ADC value was an independent predictor of *lesion volume change* and the SDS at day four, when specified instead of hyperacute relative regional perfusion parameters in the model described above (see Supplementary Table VI for details).

## Discussion

This work aimed to determine, given a certain occlusion duration, to what extent post-ischemic perfusion deficits develop after recanalization and how disease outcome is related to post-recanalization hemodynamics. Serial multiparametric MRI in a rat model of tMCAO allowed tracking of tissue fates and their subsequent demarcation into ROIs. Histological analyses confirmed that these ROIs indeed undergo changes leading to histo(patho)logical traits that grossly correspond to their classification. Four days after reperfusion, salvaged areas were nearly indistinguishable from healthy tissue. Contrarily, areas of delayed injury displayed typical patterns of infarct pathology, but with a slightly reduced infarction status compared to the lesion core. Major findings of ROI-guided analyses of cerebral perfusion after tMCAO were: 1) hyperperfusion develops after 45- or 90-min occlusion duration, whereas overt hypoperfusion is rare, 2) subacute hyperperfusion is more severe in males than in females, 3) non-ischemic tissue (appearing normal on ADC-maps during occlusion) bound for delayed injury (exhibiting abnormally prolonged T_2_ in the subacute phase) is relatively more hypoperfused shortly after recanalization, and 4) early post-recanalization hemodynamic patterns associated with lesion volume change and functional outcome.

Despite advancements made in reperfusion therapies, patient recovery rates are disappointing. For mechanical thrombectomy, the most successful reperfusion strategy to date, the number-needed-to-treat of eight shows room for improvement.^43^ IMR has long been a primary suspect for treatment failure,^7^ but hypoperfusion after recanalization was not widely observed in our study with the intraluminal filament tMCAO model, characterized by abrupt recanalization of the proximal middle cerebral artery similar to endovascular thrombectomy.^23^ This observation is in line with a recent clinical report where evidence of incomplete reperfusion, used as a proxy for IMR, was found to be highly infrequent in early perfusion-weighted images of patients in whom successful recanalization was achieved.^44^ Moreover, we recently contributed to a perfusion MRI study that showed that hyperperfusion is present in the lesion core of more than half of recanalized AIS patients after 24 h, whereas hypoperfusion was only detected in non-recanalized patients.^45^

Still, IMR has been described in different animal models of brain ischemia.^7^ Recently, neutrophils were caught in-vivo obstructing downstream microvasculature after reperfusion in mouse models of transient distal MCAO.^46,47^ Notable however, is that unlike rats,^48^ mice reperfuse poorly after 1 hour intraluminal tMCAO,^49^ possibly indicating a propensity of murine AIS models towards post-ischemic hypoperfusion, while also suggesting that such models may be unable to recapitulate the hyperperfusion syndrome found in man. Thus, successful experimental induction of IMR possibly depends on species, the nature of the obstruction (viz. the model) or treatment. The latter observation also highlights a limitation of our study, since IMR may be caused by thrombolysis-induced downstream micro-embolisms,^5^ and we did not achieve occlusion through embolism nor administered thrombolytics.

Even though we detected patches of post-ischemic hypoperfusion, IMR might not be readily demonstrable with perfusion-weighted MRI. By definition, IMR manifests at the microscopic level^7^ and may not compound to notable signal differences at the parenchymal level when it is distributed sparsely throughout the post-ischemic tissue. Moreover, gadolinium-based contrast agents dissolve in blood plasma, and may therefore be unhindered by obstructions that leave larger erythrocytes entrapped in the microvasculature.^50^ Still, partial microvascular constrictions expectedly cause flow bottlenecks, blocking erythrocytes and plasma to various degrees. The fact that normal-to-increased perfusion was observed immediately after recanalization challenges a possible notion that immediate (micro)vascular blockages found earlier in super-resolution imaging studies^46,47,50^ are rampant throughout post-ischemic tissue in this model.

Instead, post-ischemic hyperperfusion in this model was evident: it emerged rapidly, irrespective of occlusion duration and sex. Female rats demonstrated larger acute-to-subacute lesion volume reduction and an attenuated hyperperfusion response in the subacute phase after recanalization. While hyperperfusion at large has been well-established in translational imaging studies of AIS,^13^ consensus on clinical significance is still lacking due to mixed evidence, and possible sex effects remain unexplored. It is well-known that AIS outcomes are mediated by sex-dependent factors such as hormones, most prominently estrogen,^51^ but much of the molecular underpinnings are still unknown. Some explanation may be sought in recent transcriptomic^52^ and proteomic^53^ characterizations of cerebral microvessels from adult male and female Sprague-Dawley rats. It was found that, among many other sex-related differences, multiple genes and proteins involved in mitochondrial respiration were more abundant in female microvessels, while males had higher abundances of mitochondria-destructive proteins.^52,53^ While these multi-omic analyses were not performed in an experimental stroke context, it is conceivable that improved lesion outcome and an attenuated subacute hyperperfusion response in females after AIS are the result of sexual disparities in microvascular tissue function, somehow enabling (post-)ischemic tissue and microvasculature of female rats to better cope with the consequences of ischemia.

While group averages of the post-ischemic hemodynamic measures depicted normal-to-increased reperfusion, ROI-based comparisons of binned perfusion values indicated that tissue exhibits fate-specific reperfusion patterns. In terms of CBF, most voxels per ROI were classified as normally perfused early after recanalization: approximately less than a fifth was hypo- or hyperperfused. Yet areas of delayed injury seemed to be more hypoperfused compared to contralateral control areas. Areas of delayed injury appear to have been hypoperfused during MCAO, without showing signs of cytotoxic edema, and somehow failed to benefit from recanalization. At the very least, their distinct reperfusion profile could imply that either reperfusion was not effective, or the aberrant profile is an epiphenomenon of reperfusion injury.^5^

The fact that many AIS patients do not recover after successful recanalization is in urgent need of elucidation. Our linear models of experimental stroke outcome indicated that hemodynamics, dissected into three key indices of relative regional perfusion – rrCBF, rrCBV, and rrMTT – can improve the quality of a statistical model when only sex and occlusion duration are considered. Correspondingly, Luby et al.^54^ recently found that early rCBF increase in recanalized patients associated with lesion growth, thereby reproducing our findings in a clinical sample. Although the individual perfusion parameters can be important predictors of stroke outcome in their own right, combinations of post-ischemic hemodynamic indices independently modulated outcomes as well. Immediately after successful mechanical thrombectomy the autoregulatory system of ischemic tissue must adequately respond to restored perfusion pressure to provide for metabolically distressed brain cells. An inadequate response, such as increased CBF in absence of increased CBV will shorten MTT, and analogously, impair extraction of nutrients and oxygen at the level of the capillary bed.^6^ Since the neurovascular unit is responsible for cerebral autoregulation,^55^ it is tempting to speculate that the neurovascular unit fails to comply with energy demands when facing abrupt blood flow restoration due to permanent damage – possibly instigated by reperfusion itself.^5^ In line with this, preclinical MRI-studies of ischemia-reperfusion have found blunted vasomotor responses up to several days post-ischemic stroke.^19,20^ In the age of reperfusion therapies, the search for neuroprotection strategies could see a shift in goalposts: strategies that support the neurovascular unit after recanalization are highly anticipated.^56^

Consensus on clinical significance of stroke-related hyperperfusion is absent. Our multi-model inference strategy suggests that the predictive capability and direction of effect of stroke-related hyperperfusion may be parameter-specific. As such, a definition of “hyperperfusion” based on one parameter like CBF may be insufficient to predict clinical outcome comprehensively with hyperacute post-stroke hemodynamics. The parameter-specific effects can have implications for imaging studies that assess cerebral perfusion after recanalization using CBF measurements only, such as classic arterial spin labelling (ASL) methods, as other sources of hemodynamic variability pertinent to stroke outcome may be overlooked.

Proposedly, malignant “non-nutritional” hyperperfusion could be reserved for situations where CBF is increased but CBV stagnates, producing a blood transit time that is too short. “Adaptive hyperperfusion” would refer to situations where CBF is increased but CBV follows suit, ensuring MTT remains at adequate levels allowing nutrient diffusion from blood to tissue. Indeed, in our secondary analysis, we found that shorter rrMTT values correlated with lower ADC values in post-ischemic tissue immediately after recanalization, meaning that ADC values tend to normalize faster in animals with relatively longer MTT. It is known that very early ADC normalization reflects a phenomenon known as DWI lesion reversal and good clinical outcome,^12^ which we also detected in the present study (Supplementary Table VI). Conversely, the lingering ADC reduction after successful recanalization could indicate persisting metabolic failure.^57^

In conclusion, longitudinal MRI imaging of male and female rats in a tMCAO model revealed early development of persisting hyperperfusion in the post-ischemic lesion, regardless of occlusion duration, but greater in male animals. Examination of ROI-specific hyperacute reperfusion profiles suggested that non-ischemic tissue bound for secondary injury exhibits a more hypoperfused status, possibly indicative of a maladaptive hemodynamic response. Cerebral hemodynamics immediately after recanalization suggest a double-edged role for hyperperfusion, as increased rrCBV associated with beneficial outcome in terms of lesion evolution and functional outcome, while conversely, increased rrCBF and shorter rrMTT associated with detrimental outcome. Future studies could further elucidate the time-course of the hemodynamic changes and the effects on additional functional outcomes after recanalization.

## Supplemental Material

sj-pdf-1-jcb-10.1177_0271678X231208993 - Supplemental material for Hyperperfusion profiles after recanalization differentially associate with outcomes in a rat ischemic stroke model Supplemental material, sj-pdf-1-jcb-10.1177_0271678X231208993 for Hyperperfusion profiles after recanalization differentially associate with outcomes in a rat ischemic stroke model by Bart AA Franx, Geralda AF van Tilborg, Aladdin Taha, Joaquim Bobi, Annette van der Toorn, Caroline L Van Heijningen, Heleen MM van Beusekom, Ona Wu, Rick M Dijkhuizen and on behalf of the CONTRAST consortium in Journal of Cerebral Blood Flow & Metabolism
