# Supplementary material for: Hyperperfusion profiles after recanalization differentially associate with outcomes in a rat ischemic stroke model
Source: J Cereb Blood Flow Metab. 2023 Oct 24;44(2):209–23. doi: 10.1177/0271678X231208993 (PMC10993873; doi:10.1177/0271678X231208993)
Supplement: sj-pdf-1-jcb-10.1177_0271678X231208993 - Supplemental material for Hyperperfusion profiles after recanalization differentially associate with outcomes in a rat ischemic stroke model [file sj-pdf-1-jcb-10.1177_0271678X231208993.pdf]

Supplementary material

Hyperperfusion profiles after recanalization differentially predict outcome in a rat ischemic stroke model

Franx, et al.

2023-02-06

SUPPLEMENTARY FIGURES

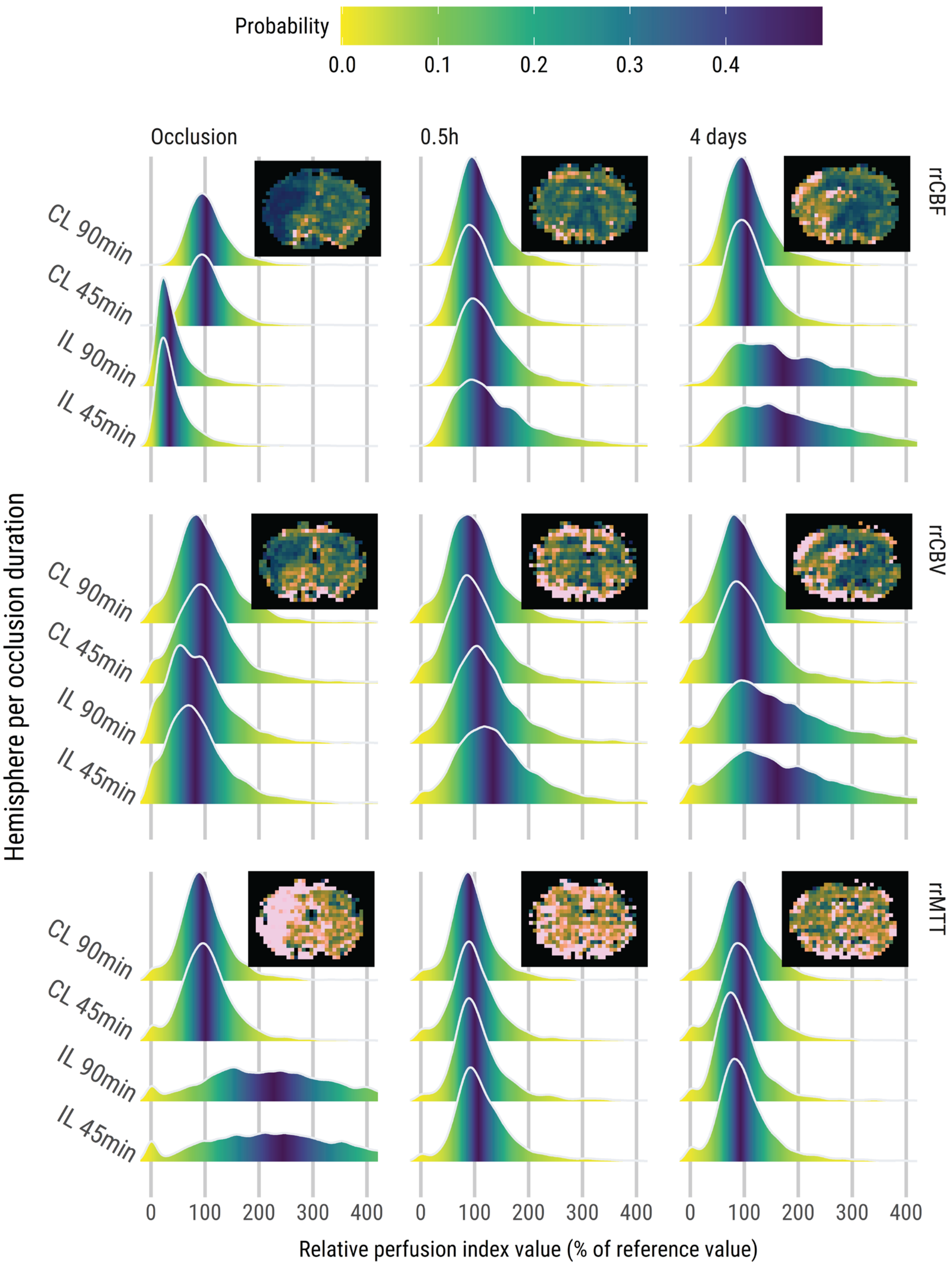

Supplementary figure I. Empirical cumulative density functions (ECDF) of the (post-)ischemic area and contralesional homologue in male rats. Voxels in the (post-)ischemic area and contralesional homologue of cerebral blood flow, cerebral blood volume and mean transit time maps (rows) during 90- or 45-min MCA occlusion, 0.5h post-recanalization and four days post-recanalization (columns) were repercentaged to a reference value, the contralateral median. ECDFs were then calculated of all repercentaged values of these areas per time point. The ECDF is associated with the empirical measure of a sample: the value of the color gradient at any specified value of the measured variable along the abscissa is the fraction of observations that are less than or equal to that specified value. Representative perfusion maps from the same male subject are displayed at the top-right corner of each cell. CL=contralesional, IL=ipsilesional, rrCBF = relative regional cerebral blood flow (rrCBF), rrCBV = relative regional cerebral blood volume, rrMTT = relative regional mean transit time.

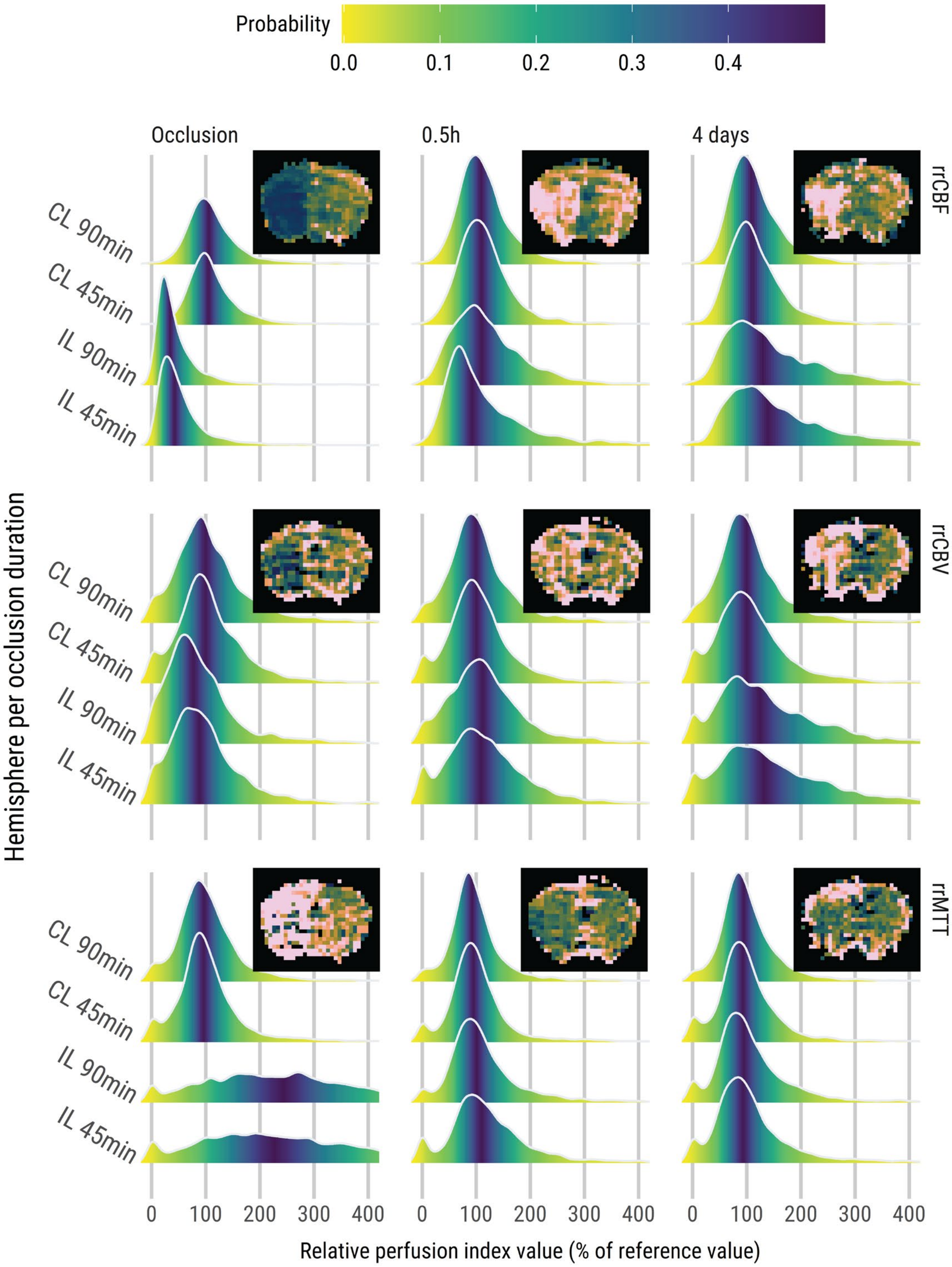
Probability
0.0
0.1
0.2
0.3
0.4
Occlusion
0.5h
4 days
rrCBF
rrCBV
rrMTT
CL 90min
CL 45min
IL 90min
IL 45min
Hemisphere per occlusion duration
0
100
200
300
400
Relative perfusion index value (% of reference value)

Supplementary figure II. Empirical cumulative density functions (ECDF) of the (post-)ischemic area and contralesional homologue in female rats. Voxels in the (post-)ischemic area and contralesional homologue of cerebral blood flow, cerebral blood volume and mean transit time maps (rows) during 90- or 45-min MCA occlusion, 0.5h post-recanalization and four days post-recanalization (columns) were repercentaged to a reference value, the contralateral median. ECDFs were then calculated of all repercentaged values of these areas per time point. The ECDF is associated with the empirical measure of a sample: the value of the color gradient at any specified value of the measured variable along the abscissa is the fraction of observations that are less than or equal to that specified value. Representative perfusion maps from the same female subject are displayed at the top-right corner of each cell. CL=contralesional, IL=ipsilesional, rrCBF = relative regional cerebral blood flow (rrCBF), rrCBV = relative regional cerebral blood volume, rrMTT = relative regional mean transit time.

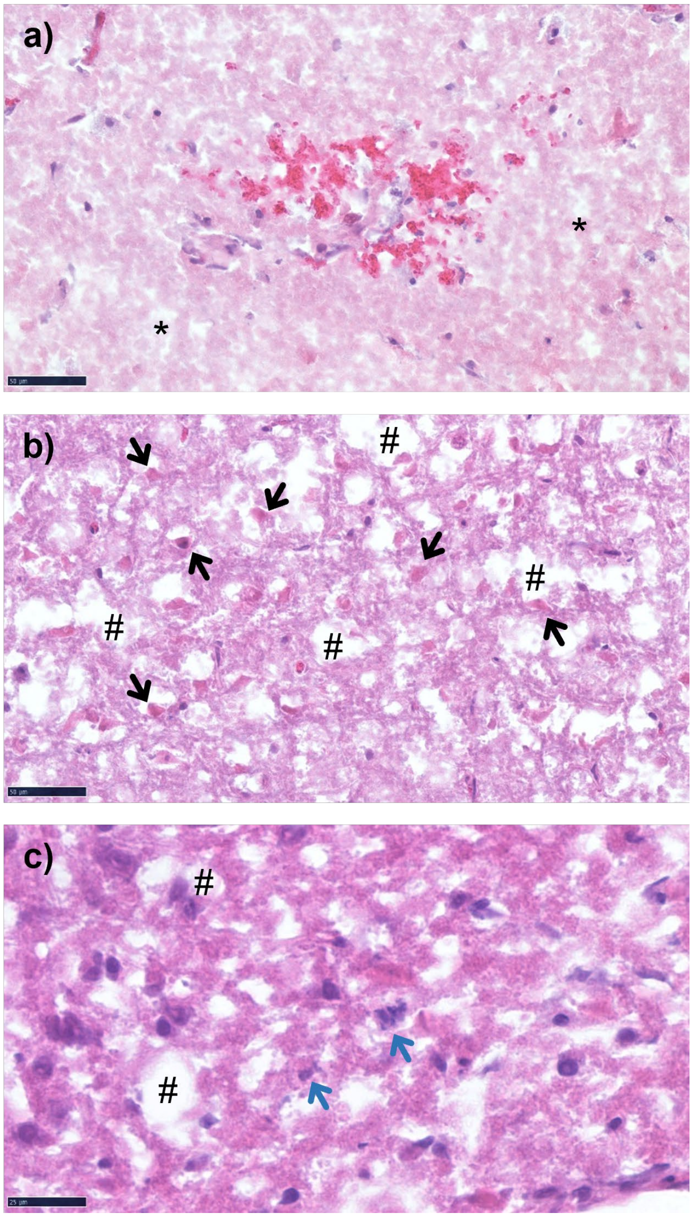

Supplementary Figure III. Histopathological features of brain infarction on hematoxylin-eosin-stained sections. **a)** Hemorrhage with abundant erythrocytes in the center of the image. Loss of both eosinophilia and nuclei can be observed in the areas marked with *. Scale bar represents 50 µm. **b)** Representative image of infarcted tissue with numerous presence of red neurons (black arrows) together with extracellular edema/vacuolization (#). Scale bar represents 50 µm. **c)** A zone with extracellular edema/vacuolization (#), in which light-blue arrows point to cells with nuclear abnormalities. Scale bar represents 25 µm.

SUPPLEMENTARY TABLES

Supplementary Table I – Sensorimotor deficit score (SDS) test items

**a)**	**Gait disturbances in open area during one minute (0-5)**	0. Straight walking;<br>1. Walking towards paretic side;<br>2. Alternate circling and walking straight;<br>3. Alternate circling and walking towards paretic side;<br>4. Circling and/or other gait disturbance (backing, crawling, walking on digits);<br>5. Constant circling towards paretic side.
**b)**	**Motility during one minute (0-5)**	0. Normal exploratory behavior;<br>1. Slightly reduced exploratory behavior;<br>2. Moving limbs without proceeding;<br>3. Moving only to stimuli;<br>4. Unresponsive to stimuli, with normal muscle tone;<br>5. Severely decreased tone, premortal signs.
**c)**	**Lateral resistance (0-2)**	0. When pushed lateral, resistance equilateral;<br>1. When pushed lateral, less resistance in paretic direction;<br>2. When pushed lateral, no resistance in paretic direction.
**d)**	**Forelimb placing tests**	**I. Placement response when the contralateral vibrissae are stimulated (0-2)**<br>0. Normal;<br>1. Weak (tries but doesn't manage to put paw on table);<br>2. Absent (does not respond to stimulation).<br>**II. Placement response when the ipsilateral vibrissae are stimulated (0-2)**<br>0. Normal;<br>1. Weak (tries but doesn't manage to put paw on table);<br>2. Absent (does not respond to stimulation).
**e)**	**Forelimb reflexes after touching**	**I. Symmetry of grasping reflex of forepaw onto a rod when slightly touched (0-1)**<br>0. Symmetrical (also applies if both sides function and alternate when trying multiple times)<br>1. Asymmetrical (one paw doesn't go to the rod when touched)<br>**II. Symmetry of strength, releasing tension when withdrawn for wire or rod (0-1)**<br>0. Symmetrical;<br>1. Asymmetrical (when hanging from a rod 1 paw releases)
**f)**	**Postural signs (when held by tail base)**	**I. Degree of thorax twisting to one side (0-2)**<br>0. No thorax twisting (hanging vertically);<br>1. Light thorax twisting;<br>2. Heavy thorax twisting.<br>**II. Degree of forelimb flexion towards contra-lateral side (0-2)**<br>0. No forelimb flexion;<br>1. Light forelimb flexion;<br>2. Heavy forelimb flexion.

Supplementary Table II – Acute ischemic tissue volume, subacute infarct volume and region-of-interest volumes, expressed as hemispheric volume fraction and % of acute ischemic lesion volume

	Female, 45min (N=9)	Male, 45min (N=12)	Female, 90min (N=10)	Male, 90min (N=11)
**Acute ischemic volume**				
Mean (SD)	0.203 (0.094)	0.180 (0.107)	0.141 (0.082)	0.151 (0.102)
Range	0.063 - 0.362	0.041 - 0.363	0.041 - 0.267	0.046 - 0.345
**Subacute infarct volume**				
Mean (SD)	0.123 (0.083)	0.175 (0.142)	0.130 (0.106)	0.171 (0.150)
Range	0.029 - 0.297	0.010 - 0.452	0.013 - 0.316	0.028 - 0.430
**Lesion core**				
Mean (SD)	0.107 (0.071)	0.143 (0.107)	0.106 (0.084)	0.126 (0.106)
Range	0.024 - 0.251	0.010 - 0.315	0.012 - 0.249	0.023 - 0.319
**Salvageable tissue**				
Mean (SD)	0.096 (0.060)	0.037 (0.020)	0.035 (0.013)	0.025 (0.012)
Range	0.029 - 0.201	0.006 - 0.069	0.018 - 0.057	0.012 - 0.047
**Delayed injury**				
Mean (SD)	0.015 (0.014)	0.033 (0.042)	0.024 (0.023)	0.045 (0.055)
Range	0.004 - 0.046	0.000 - 0.137	0.001 - 0.067	0.005 - 0.181
**Rel. subacute infarct volume (%)**				
Mean (SD)	57.4 (23.2)	85.1 (33.6)	78.6 (31.3)	99.1 (35.3)
Range	29.9 - 105.9	24.0 - 139.0	32.1 - 118.2	49.8 - 174.1
**Rel. lesion core (%)**				
Mean (SD)	50.2 (19.8)	69.6 (22.3)	64.7 (22.4)	74.0 (18.9)
Range	26.5 - 89.5	23.8 - 97.9	28.5 - 93.1	41.0 - 94.8
**Rel. salvageable tissue (%)**				
Mean (SD)	49.8 (19.8)	30.4 (22.3)	35.3 (22.4)	26.0 (19.0)
Range	10.5 - 73.5	2.1 - 76.2	6.9 - 71.5	5.2 - 59.4
**Rel. delayed injury (%)**				
Mean (SD)	7.2 (4.1)	15.5 (14.0)	13.9 (10.0)	25.1 (21.2)
Range	2.9 - 16.4	0.2 - 42.1	3.2 - 28.5	7.4 - 80.9

Supplementary Table III – Summary of main effects per perfusion index from linear mixed model analyses

Term	F	df	Residual df	*p*
		Cerebral blood flow		
tMCAO (90min)	0.29	1	37.44	0.59
Sex (M)	***8.44***	***1***	***37.25***	***0.006***
Time	***149.24***	***2***	***73.05***	***< 0.001***
tMCAO:Sex	0.11	1	37.27	0.74
tMCAO:Time	0.17	2	73.24	0.85
Sex:Time	***3.51***	***2***	***73.1***	***0.035***
tMCAO:Sex:Time	1.02	2	73.15	0.37
		Cerebral blood volume		
tMCAO (90min)	2.01	1	37.34	0.16
Sex (M)	***9.7***	***1***	***37.14***	***0.004***
Time	***66.2***	***2***	***73.21***	***< 0.001***
tMCAO:Sex	0.07	1	37.18	0.79
tMCAO:Time	0.87	2	73.4	0.42
Sex:Time	1.55	2	73.24	0.22
tMCAO:Sex:Time	0.25	2	73.29	0.78
		Mean transit time		
tMCAO (90min)	0.48	1	37.25	0.49
Sex (M)	0.33	1	37.05	0.57
Time	***279.94***	***2***	***73.34***	***< 0.001***
tMCAO:Sex	0.02	1	37.09	0.89
tMCAO:Time	0.21	2	73.52	0.81
Sex:Time	0.58	2	73.36	0.56
tMCAO:Sex:Time	0.28	2	73.4	0.76

Abbreviation:

tMCAO: transient middle cerebral artery occlusion

Note: residual degrees of freedom (df) are Kenward-Roger approximations

Supplementary table IV – Post-hoc analyses of cerebral perfusion indices before and after recanalization

Sex	Time	Contrast	Estimate	Effect size	SE	Residual df	t	*p*
				Cerebral blood flow				
.	1	M-F	0.58	0.02	9.09	106.82	0.06	> 0.99
.	2	M-F	17.36	0.63	9.09	106.82	1.91	0.41
.	***3***	***M-F***	***33.56***	***1.23***	***9.31***	***107.25***	***3.61***	***0.003***
F	***.***	***2-1***	***53.48***	***1.95***	***8.9***	***71.57***	***6.01***	***< 0.001***
F	***.***	***3-2***	***34.91***	***1.27***	***8.9***	***71.57***	***3.92***	***0.001***
M	***.***	***2-1***	***70.26***	***2.57***	***8.29***	***71.57***	***8.48***	***< 0.001***
M	***.***	***3-2***	***51.11***	***1.87***	***8.56***	***76.65***	***5.97***	***< 0.001***
				Cerebral blood volume				
.	1	M-F	3.81	0.17	7.25	108.57	0.53	> 0.99
.	2	M-F	16.75	0.74	7.25	108.57	2.31	0.16
.	***3***	***M-F***	***20.88***	***0.93***	***7.43***	***108.66***	***2.81***	***0.041***
F	***.***	***2-1***	***22.84***	***1.01***	***7.32***	***71.67***	***3.12***	***0.018***
F	***.***	***3-2***	***26.17***	***1.16***	***7.32***	***71.67***	***3.57***	***0.004***
M	***.***	***2-1***	***35.77***	***1.59***	***6.83***	***71.67***	***5.24***	***< 0.001***
M	***.***	***3-2***	***30.3***	***1.34***	***7.02***	***76.89***	***4.31***	***< 0.001***
				Mean transit time				
.	1	M-F	5.15	0.17	9.67	109	0.53	> 0.99
.	2	M-F	-6.9	-0.22	9.67	109	-0.71	> 0.99
.	3	M-F	-8.27	-0.27	9.91	109	-0.83	> 0.99
F	***.***	***2-1***	***-125.86***	***-4.09***	***10.01***	***71.77***	***-12.58***	***< 0.001***
F	.	3-2	-13.92	-0.45	10.01	71.77	-1.39	> 0.99
M	***.***	***2-1***	***-137.91***	***-4.48***	***9.32***	***71.77***	***-14.79***	***< 0.001***
M	.	3-2	-15.28	-0.5	9.57	77.06	-1.6	0.8

Abbreviations:

M: male; F: female. Times 1, 2 and 3 indicate relative experimental times: during MCAO (1), 0.5h post-recanalization (2) and 4 days post-recanalization (3)

Note: residual degrees of freedom (df) are Kenward-Roger approximations

Supplementary table V – Coefficients and performance measures for models with different configurations of perfusion parameters regressed against sensorimotor deficit score and ranked by performance (AICc)

		Models (ranking)					
		rrMTT<br>(1)	¬*DSC-PWI*<br>(2)	rrCBF & rrCBV<br>(3)	rrCBV<br>(4)	rrCBF<br>(5)	*NULL*<br>(6)
**Model coefficients (standardized β (±95 CI))**	rrCBF			**0.3***		-0.01	
				**(0.01, 0.6)**		(-0.2, 0.1)	
	rrCBV			**-0.4***	-0.1		
				**(-0.7, -0.1)**	(-0.3, 0.04)		
	rrMTT	**-0.2***					
		**(-0.3, -0.01)**					
	tMCAO (90min.)	0.4$^{\#}$	**0.5***	**0.5***	**0.5***	**0.5***	
		(-0.004, 0.8)	**(0.1, 0.9)**	**(0.1, 0.9)**	**(0.1, 0.9)**	**(0.1, 0.9)**	
	Sex (M)	0.1	0.2	0.3	0.3	0.2	
		(-0.3, 0.5)	(-0.2, 0.6)	(-0.1, 0.7)	(-0.1, 0.7)	(-0.2, 0.6)	
	Acute ischemic volume	**0.3****	**0.4****	**0.3****	**0.4****	**0.4****	
		**(0.1, 0.5)**	**(0.2, 0.6)**	**(0.1, 0.5)**	**(0.2, 0.6)**	**(0.2, 0.6)**	
	Subcortical lesion	0.3	0.3	0.2	0.2	0.3	
		(-0.2, 0.7)	(-0.1, 0.8)	(-0.3, 0.7)	(-0.2, 0.7)	(-0.1, 0.8)	
	Diencephalic lesion	**-1.0****	**-1.0****	**-1.1****	**-1.1****	**-1.0****	
		**(-1.7, -0.4)**	**(-1.7, -0.4)**	**(-1.8, -0.5)**	**(-1.7, -0.5)**	**(-1.7, -0.4)**	
	tMCAO:Sex	-0.3	-0.3	-0.4	-0.4	-0.3	
		(-0.8, 0.3)	(-0.8, 0.2)	(-0.9, 0.1)	(-0.9, 0.1)	(-0.8, 0.2)	

Supplementary table V (continued)

**Model performance**	AICc	228	229.2	229.2	230	232.2	278.7
	X	4.33	0	6.37	2.26	0.03	
	p ($>X_{\neg DSC\text{-}PWI}$)	0.04	1	0.04	0.13	0.85	
	RMSE	3	3.1	2.9	3	3.1	4.3
	Residual df	33	34	32	33	33	40
	Adjusted R^2 (Nagelkerke)	0.84	0.82	0.85	0.83	0.82	0

Note: Models were tested against the base model without perfusion indices (¬DSC-PWI), which included only tMCAO, sex and nuisance variables

#$p < .10$; *$p < .05$; **$p < .01$

Abbreviations: tMCAO: transient middle cerebral artery occlusion. rrCBF: relative regional cerebral blood flow. rrCBV: relative regional cerebral blood volume. rrMTT: relative regional mean transit time. AICc: corrected Akaike information criterion, RMSE: root mean square error.

Supplementary table VI – Acute post-ischemic ADC (instead of relative regional perfusion), occlusion duration, sex and control variables regressed against lesion volume change or SDS

	Dependent variable:	
	Lesion volume change	SDS
Assumed distribution of dependent variable:	*Normal*	*Poisson*
	Unstandardized β (±95 CI)	Unstandardized β (±95 CI)
0.5h ADC	**-15.31** (-25.33, -5.29)**	**-0.21** (-0.38, -0.05)**
tMCAO (90min.)	5.55 (-21.12, 32.21)	0.10 (-0.39, 0.59)
Sex (M)	16.69 (-4.19, 37.58)	-0.002 (-0.40, 0.40)
Acute ischemic volume	6.19 (-6.12, 18.50)	**0.27* (0.05, 0.49)**
Subcortical lesion	-27.83# (-52.93, -2.72)	0.22 (-0.24, 0.69)
Diencephalic lesion	**-27.96* (-53.27, -2.65)**	**-1.01** (-1.64, -0.38)**
tMCAO:Sex	-0.62 (-28.97, 27.72)	-0.16 (-0.69, 0.36)
F	9.25	
Log Likelihood		-105.97
RMSE	19.6	2.97
Residual df	34	34
Adjusted R^2	0.58	
Adjusted R^2 (Nagelkerke)		0.87

Abbreviations: SDS: sensorimotor deficit score. OLS: ordinary least-squares. CI: confidence interval. ADC: apparent diffusion coefficient. RMSE: root mean square error.

#p < .10; *p < .05; **p < .01
